# Application of *Pontentilla Anserine*, *Polygonum aviculare* and *Rumex Crispus* Mixture Extracts in A Rabbit Model with Experimentally Induced *E. coli* Infection

**DOI:** 10.3390/ani9100774

**Published:** 2019-10-09

**Authors:** Robert Kupczyński, Antoni Szumny, Michał Bednarski, Tomasz Piasecki, Kinga Śpitalniak-Bajerska, Adam Roman

**Affiliations:** 1 Department of Environment, Animal Hygiene and Welfare, Wrocław University of Environmental and Life Sciences, 51-630 Wrocław, Poland; kinga.spitalniak-bajerska@upwr.edu.pl (K.Ś.-B.); adam.roman@upwr.edu.pl (A.R.); 2 Department of Chemistry, Wroclaw University of Environmental and Life Science, 50-375 Wrocław, Poland; antoni.szumny@upwr.edu.pl; 3 Department of Epizootiology with Clinic of Birds and Exotic Animals, Wroclaw University of Environmental and Life Science, 50-366 Wrocław, Poland; michal.bednarski@upwr.edu.pl (M.B.); tomasz.piasecki@upwr.edu.pl (T.P.)

**Keywords:** rabbit, herbs, *E. coli*, diarrhea, blood parameters

## Abstract

**Simple Summary:**

The beneficial effect of herbs on production parameters, quality of products of animal origin, and animal health was demonstrated in numerous studies. This study aimed to determine an effect of herbal mixture on blood parameters and the anti-colibacteriosis efficacy. The rabbit model used during the experimental infection with *E. coli* indicates a clear effect of *Rumex crispus* L., *Pontentilla anserine*, *Polygonum aviculare*, whose activity involves the reduction of colonization of basically all sections of the intestines. The use of herbs in rabbits can control the activity of intestinal microbial community. Administration of a mixture of herbs to feed reduced the number of *E. coli* in cecum more than supplementation into water after experimental infection. The plant extracts may be a good alternative to antibiotic treatment.

**Abstract:**

The study evaluated the anti-colibacteriosis efficacy of herbs in experimental infection by rabbit pathogenic strain of *E. coli* O103 *eae*^+^. It also studied the effects of herbal mixture added to feed or water on blood parameters. This animal model was used since some *E. coli* strains pathogenic for rabbits are similar to the strains that are pathogenic to humans. The components of herbal extracts were *Rumex crispus*, *Pontentilla anserine*, and *Polygonum aviculare*. Supplementation was carried out in water (ExpW group) or feed (ExpF group), and four weeks later the animals were infected with the *E. coli* O103 *eae*^+^ strain. The administration of herbs increased the mean concentration of total protein and serum albumin (*p* < 0.01) without causing disturbances of electrolyte and acid-base balance. The highest total antioxidant capacity (TAS) value (*p* < 0.01) was observed in the ExpF group. The administration of a mixture of herbs and feed caused more reduction in the number of *E. coli* in cecum than supplementation into water after an experimental infection. The herbs applied in rabbits did not harm the secretory functions of liver, electrolyte, and acid-base balance of the blood. The application of the tested herbal mixtures can control the activity of the intestinal microbial community.

## 1. Introduction

Biologically active compounds present in the plants may influence the microorganism. Yellow dock (*Rumex crispus* L.) is a weed plant whose roots and green parts are moderately used in traditional and veterinary medicine [1]. However, there is a lack of clinical research assessing the effects of yellow dock, and rigorous randomized controlled clinical trials are still stated to possess slightly purgative and cholagogue properties [2]. According to European Pharmacopoeia, it is still being used for chronic skin disease, obstructive jaundice, constipation, and specifically psoriasis with constipation. Biological activity of its roots is associated with antraquinone-type compounds, e.g., emodin, hrysophanol, nepodin or physcion and their corresponding glycosidies. Slightly antimicrobial activity against *Staphylococcus aureus* and *Bacillus subtilis* of water extracts was assessed [3]. Methanolic extracts of the aerial part of the yellow dock from Eastern Anatolia region of Turkey possessed very strong activity against several pathogenic bacteria, e.g., *Salmonella typhimurium*, *Vibrio cholerae*, *Yersinia frederiksenii*, or *Yersinia pseudotuberculosis* [4]. It should be underlined that the use of yellow dock so far was limited to roots or the green part. In our work we applied for the first time the extracts obtained from inflorescences to control diseases in rabbits. In our work we applied for the first time the extracts obtained from inflorescences, which are in folk tradition in Central European countries applied to control bee diseases.

The aerial part of the common knotgrass (*Polygonum aviculare* L.) is a safe and effective astringent and diuretic agent that is used mainly in the treatment of complaints such as dysentery and hemorrhoids. It is also traditionally used in the treatment of pulmonary and it is attributed to the anthelmintic, cardiotonic, astringent, cholagogue, diuretic, and febrifuge activity. It was formerly widely used as an astringent both internally and externally in the treatment of wounds, bleeding, piles, and diarrhea. The diuretic properties make it useful in folk medicine for removing kidney stones. In traditional medicine, water macerates are also used to increase the amount of urine to achieve flushing of the urinary tract or as an adjuvant in minor urinary complaints. Some sources also describe its application for the symptomatic treatment of minor inflammations in the mouth or the throat [5].

Several pharmacopoeias contain monographs on *Potentilla* species, e.g., European Pharmacopoeia [6]. *Tormentillae tinctura* (*Potentilla erecta* rhizomes) is official in the Ph. Eur. (drug-to-extract ratio 1:5, solvent: ethanol 70% (v/v)), contains a minimum of 1.5% (m/m) of tannins, expressed as pyrogallol equivalent. Biological activity is assessed with several triterpenoid saponins as corresponding aglycones, e.g., ursolic, tormentic acids or isomers of amyrins or tannins [7,8]. Traditional use of tormentil in the treatment of colitis ulcerosa was proven in a clinical study [9]. The antisecretory activity of silwerweed extracts was proven in a clinical trial [10]. Synowiec et al. [11] proved the strong antimicrobial effect of *Potentilla erecta* water extracts against *Staphylococcus aureus* and *Candida lipolitica* strains and catechines were selected as active fraction.

Diarrheas constitute a major clinical problem of many farm animals [12,13]. One of the important causes of diarrhea in animals is the pathogenic strains of *E. coli*. Due to their virulence factors, these strains in case of rabbits are included in the enteropathogenic *E. coli* group (EPEC) and are called rabbit pathogenic *E. coli* (RPEC). *E. coli* strains pathogenic for rabbits are similar to the strains that are pathogenic to humans [14]. The main factor in the pathogenicity of enteropathogenic *E. coli* is intimin. It is an adhesive protein of a pathogenic strain that allows close adherence of bacterial cells to the intestinal villi and causes obstruction of their structure and intestine inflammation [12,14]. It is possible that an Esp-dependent but intimin-independent effect triggered by certain EPEC strains is important in vivo and could account, for instance, for the persistent diarrhea often associated with human EPEC infections [15].

At present, the treatment and control of colibacilosis in rabbits is mainly based on antibiotic therapy. This results in the widespread use of antibiotics and hence the emergence of resistance among bacterial strains [16]. Currently, the phenomenon of resistance among bacterial strains is a serious problem in the treatment of both humans and animals and is widely publicized, inter alia by the European Medicines Agency (EMA) and EFSA [17]. Having the above in mind, there is a growing need for effective and alternative methods, such as a vaccine or plant extracts, to cope with these problems and to reduce the use of antibiotics. About 65% of antibacterial drugs approved in 1981–2010 were natural products or their semi-synthetic derivatives [18]. Standardized plant extracts can be used as feed additives or medicinal fodders. Herbs and natural substances remain the subject of intense research to find, on the one hand, new compounds, and on the other, their application in therapy due to antipathogenic activity, affecting production indices or, for example, the immune system. Taking into account the antimicrobial activity of extracts or substances of herbal origin, their use in nutrition may reduce susceptibility to infection and thus the amount of antibiotics used. The beneficial effects of herbs on production parameters, quality of animal origin products, and animal health were found using them as dietary additive for rabbits [19]. Diarrhea and mortality resulting from infections with EPEC are of major economic importance in the rabbit meat industry. An effect of ethanolic extract of propolis (EEP) and herbal mixture on chronic diarrhea symptoms in rabbits was compared in our previous study [20]. The application of herbal mixture or EEP to drinking water for ten days resulted in accelerated recovery. The present study evaluated the anti-colibacteriosis efficacy of herbs in experimental infection by rabbit pathogenic strain of *E. coli* O103 *eae*^+^. It also studied the effects of herbal mixture added to feed or water on blood parameters.

## 2. Materials and Methods:

### 2.1. Plant and Extraction Conditions

The components of herbal extract were common silverweed *(Argentina anserine*, herb)*,* as well as common silverweed (*Polygonum aviculare,* herb) infusions were prepared from commercially produced loose leaves kindly provided by Herbapol (Wrocław, Poland). *Rumex* *crispus* L. inflorescences were authenticated by Przemysław Bąbelewski from Wrocław University of Environmental and Life Sciences. The amounts and method of herbal extract preparation were determined in previous in vivo study [21]. About 10 g of powdered dried herbs mixture (aerial part of common knotgrass, common silverweed, and inflorescences of yellow dock in rate 1:1:1) was macerated in 100 mL of distilled water at 80 °C during 30 min. The dry mass content in extracts was measured by preliminary concentrated on vacuum rotary evaporator and next freeze-drying, finally assessed on 3.6% (m/v).

### 2.2. Chemical Assessment of Bioactive Compounds

Due to fact that for biological experiment three-compounds mixture of herbs, which are multiple complex of compounds, we decided to perform chemical analysis for water freeze-dried residue of each herb (3.3 g in 100 mL of distilled water within at 80 °C during 30 min) separately macerated. 

#### 2.2.1. Total Phenolics 

Total phenolic compounds (TPC) were measured for powdered plant freeze-dried macerates according to literature [22,23,24]. The TPC contents were measured using a Folin-Ciocalteu assay. The results were expressed as mg equivalent of gallic acid per mL of macerate, according to calibration curves.

#### 2.2.2. Proanthocyanidins Measurement

For analysis of total amount of proanthocyanidins (PAC) butanol-acid test, according to Lincheva et al. [22]. In brief, 10 mL of HCl-*n*BuOH stock solution (4 mL of 36% hydrochloric acid mixed with 96 mL of *n*BuOH), around 50 mg of plant (macerate and 0.15 mL solution of NH_4_Fe(SO_4_)_2_·12 H_2_O in 2M HCl was stirred in a glycerine bath within 30 min in temperature 95 °C. Concentrations of PAC were measured on the basis of formed colour complex (550 nm). Proanthocyanidins content were calculated as leucocyanidin equivalent presented in 1 mL of plant water extract.

#### 2.2.3. Total Tannins Assay Phenolic Browning Assay

A spectrophotometric assay of the rate of browning of low-molecular-weight phenolic compounds was adapted to measure the browning of tannins, according to protocol proposed by Lincheva et al. [22]. The samples 50 mg of freeze-dried plant macerate (diluted 1:2 with 70% ethanol) was dissolved in 7.7 mL pH 10 buffer (5 mM Na_2_CO_3_: 5 mM NaHCO_3_ in ratio 6:4). Absorbance was measured at 415 nm, beginning at 15 s after the addition of the sample. 

Total tannins were assessed according to the spectrophotometric method [25]. Briefly, 50 mg of plant freeze-dried macerate was dissolved in 6.5 mL of pH 10 sodium bicarbonate buffer and absorbance was measured every 60 s in time within 10 min. As a standard, galloyl glucose was used. 

#### 2.2.4. Antraquinones Measurment

Quantification of anthraquinones in *R. crispus* was based on protocol presented by Sakulpanich et al. [26]. In brief, powdered plant material was shaken of distilled water. After 24 h solution was centrifuged in 9000 rpm and supernatant (with pH 2.0 adjusted by 0.5 M HCl) was extracted three times with chloroform. Addition of FeCl_3_ and next acidification with 2M HCl gave fraction enriched with anthraquinone aglycones. Extraction to Et_2_O, and drying over magnesium sulphate gave finally compounds, quantified on 515 nm on spectrophotometer using emodin (purity > 95%, supplied by UQF—United Quantum Factory Poland) as standard for calibration curve.

### 2.3. Chromatographical Analyses

#### 2.3.1. Low-Molecular Polar Fraction Analysis

Carbohydrates and water-soluble low-molecular compounds were assessed by derivatization with N,O-Bis (trimethylsilyl)trifluoroacetamide (BSTFA) silylation approach on GC-MS (Shimadzu QP 2020, Shimadzu, Kyoto, Japan). Protocol was based according to Andrews [27]. Separation was achieved using Zebron ZB-5 capillary column with a length of 30 m, inner diameter of 0.25 mm, and film thickness of 0.25 μm (Phenomenex, Torrance, CA, USA). Profile of fatty acids, sterols, and volatile compounds of *R. crispus* was added to supplementary data (Tables S1 and S2). According to the best of our knowledge, there is no published information concerning the chemical composition of *R. crispus* inflorescence. 

#### 2.3.2. FAME Profile

The lipid fraction was obtained according to the previously described method [28]. In the next step the extracted unpolar fraction, about 50 mg, was saponified (10 min at 75 °C) with a 5 mL 0.5 M solution of KOH/MeOH and subjected to methylation (10 min at 75 °C) in 5 mL 14% (v/v) BF_3_/MeOH (Sigma-Aldrich, St. Louis, MO, USA). Then, water was added to reaction and the methyl esters of fatty acids were extracted with 20 mL of hexane (UQF Wrocław, Poland), washed with 10 mL 10% sodium bicarbonate (UQF Wrocław, Poland) and dried over unhydrous sodium sulphate. The organic phase was evaporated under reduced pressure and stored in –27 °C until chromatographical analysis. FAME profile was assessed using gas chromatograph coupled with a mass spectrometer (Shimadzu GCMS QP 2020, Shimadzu, Kyoto, Japan). Separation was achieved using Zebron ZB-WAX capillary column with a length of 30 m, inner diameter of 0.25 mm, and film thickness of 0.25 μm (Phenomenex, Torrance, CA, USA). The GC-MS analysis was according to the following parameters: Scanning was performed from 50 to 400 m/z in electronic impact (EI) at 70 eV, mode at 5 scan s−1 mode. Analyses were carried out using helium as carrier gas at a flow rate of 1.0 mL min−1 in a split ratio of 1:10 and the following program: (a) 40 °C for 3 min; (b) rate of 5.0 °C min−1 from 40 to 160 °C; (c) rate of 30 °C min−1 from 160 to 280 °C. Injector was held at 220 °C, respectively. Compounds were identified by using three different analytical methods that compare: (i) Retention times with authentic chemicals (Supelco 37 Component FAME Mix); (ii) obtained mass spectra, with available library (Willey NIST 17, match index >90%). 

### 2.4. Sterol Profile

Inflorescences of R. crispus were ground by using a mill. Basic hydrolysis was applied to collect total phytosterols from samples. Protocol was based according to Hua et al. [29]. Briefly, 1.0 g of each sample was accurately weighed in a screw cap glass tube, and 0.5 mL of cholesterol in MeOH (0.5 mg/mL, internal standard) was added and mixed. Two mL of 0.5 M MeOH/KOH was added to the sample. After heating at 60 °C for 30 min, the sample tubes were cooled under running tap water. Next, the non-polar compounds were extracted to 5 mL of cyclohexane, washed with sodium bicarbonate and brine, and finally dried over anhydrous Na_2_SO_4_. After evaporation of organic solvent, 0.2 mL of a 1:1 mixture of pyridine and BSTFA + 1% TMCS was added in the capped tube or vial. The reaction was followed about 30 min in 60 °C. TMS-derivatives profile was assessed using gas chromatograph coupled with a mass spectrometer (Shimadzu GCMS QP 2020, Shimadzu, Kyoto, Japan). Separation was achieved using Zebron ZB-5 capillary column (with a length of 30 m, inner diameter of 0.25 mm, and film thickness of 0.25 μm (Phenomenex, Torrance, CA, USA). The GC-MS analysis was according to the following parameters: Scanning was performed from 50 to 500 m/z in electronic impact (EI) at 70 eV, mode at 5 scan s^−1^ mode. Analyses were carried out using helium as carrier gas at a flow rate of 1.0 mL min^−1^ in a split ratio of 1:10 and the following program: (a) 120 °C for 3 min; (b) rate of 5.0 °C min^−1^ from 120 to 160 °C; (c) rate of 15 °C min^−1^ from 160 to 300 °C and held 5 min. Injector was set on at 250 °C, respectively 2.5. 

### 2.5. Volatile Compounds Profile

Analysis of volatile compounds was performed according to methodology Łyczko et al. [30]. The HS-SPME approach was applied for analysis. All compounds were analyzed on the basis of comparison of experimentally obtained compound mass spectra with mass spectra available in the NIST17 database. For all compounds experimental retention indices were compared with literature values [31].

### 2.6. Animals and Experimental Design

Hybrid rabbits (Hyplus line) aged 50 ± 3 days were used in this experiment. All animals were clinically healthy at the beginning of the experiment and free from any disease including coccidiosis. The animals were housed in vivarium, two animals per cage under a temperature of 20 ± 2 °C, and a 12:12 light: dark cycle (lights on 06:00 am). 

After a period of adaptation (14 day), the rabbits were randomly divided into three groups (n = 12): Group I—control, fed standard complete diet, Group II—experimental, rabbits were fed the standard diet and received herbal extract instead in drinking water (Group ExpW), Group III— experimental fed complete diet with the addition of herbal extract (ExpF). Powdered dried herbs mixture (1:1:1) for rabbits was prepared fresh each day by macerating herbs in water (10 mL/100 mL). In the group ExpF, herbal extract was every day applied on the fodder (spraying method) in the amount of 10 mL/100 g. Throughout the study, the rabbits received ad libitum each diet and had free access to water or herbal aqueous extract. Commercial diet (pellets, De Heus Poland) for growing rabbits was containing: 15.8% crude protein, 4.2% crude fat, and 18.50% crude fiber (on DM basis). Chemical analysis of diets were determined according to the AOAC method [32].

After the initial period of treatment with herbs extracts (30 day), two subgroups (n = 6) were distinguished from each group (ExpW and ExpF): The first subgroup was not infected and the second was infected with the pathogenic strain of *E. coli*. All subgroups of rabbits were kept completely isolated from each other and the treatment according to the above division was applied to the end of the whole experiment. 

Animals were observed daily to record health status of the animals checking for the presence of diarrhea, depression, sneezing, coughing. Food and drink consumption was recorded daily. Rabbits were weighed in the morning before feeding at the beginning of the experiment and after that every second day to the end of experiment. All experiments were performed in accordance with the protocol approved by the Animal Experimentation Committee of the 2nd Local Ethical Committee for Experiments on Animals in Wrocław, Poland (No. 156).

### 2.7. Blood Analyses

Blood samples from all animals were collected from the central ear artery at the beginning and after 14 and 30 days of treatment (before experimental infection). Blood was collected to sterile test tubes: For serum (2 mL, Sarstedt, Nümbrecht, Germany), to the tubes with anticoagulant—EDTA-K3 (0.5 mL, Sarstedt, Nümbrecht, Germany), and 0.2 mL heparinized capillary (Microcaps, Idexx Laboratories Inc., Westbrook, ME, USA) for arterial blood gas examinations (analysis were made immediately after blood collection). The blood samples for serum were centrifuged for 15 min at 3000× g at room temperature (2 h after samples collection), and the serum samples were frozen until the analyses (−20 °C). Hematological parameters analysis were performed using ABC Vet analyzer (HoribaABX, Montpellier, France), taking into account parameters like: Red blood cell (RBC), white blood cell (WBC), platelets (PLT), hemoglobin (HGB), hematocrit (Hct), mean corpuscular volume (MCV), mean corpuscular hemoglobin (MCH) and mean corpuscular hemoglobin concentration (MCHC). Acid-base parameters were determined using a VetStat analyzer (Idexx Laboratories Inc., Westbrook, ME, USA). 

The laboratory analyses of blood serum were done using Pentra 400 biochemical analyzer Horiba ABX (Horiba ABX, Montpellier, France) using reagents from Horiba ABX and Randox (Crumlin, Dunlin, Ireland). The following parameters were estimated:

— glucose by oxidase method;
— lactic acid (LA) via the colorimetric method;
— total protein and albumin via colorimetric method;
— total cholesterol by enzymatic method;
— total bilirubin concentration via colorimetric method;
— aspartate aminotransferase (AST) and γ -glutamyltransferase (GGT) enzyme activity via kinetic method at 37 °C;

The parameters determining the anti-oxidative status were also determined:

— total antioxidant capacity (TAS) in serum by colorimetric method based on ABTS (2,2’-azine-di [3-ethylbenzothiazoline sulphate]) method with peroxidase,
— glutathione peroxidase (GPx) in whole blood using enzymatic method, Randox reagents Ransel RS (Crumlin, UK).

### 2.8. Experimental Infections

Experimental infections were carried out with *E. coli* strains no 84/2011. The strain was isolated from massive outbreaks of diarrhea case in weaned rabbit. The strain was characterized according to Swennes et al. [33] as O103, eaeA^+^ positive. Isolated strain was kept at −80 °C in Microbank Storage Boxes (Pro-Lab Diagnostic, Canada). The infection was made after 30 days of herbs administration. The inoculations of rabbits were done according to the methods described by Boullier et al. [34]. All rabbits after the infection were each day examined clinically, dead individuals were sectioned (pathological examination) and microbiologically tested. On day seven after the infection, all the surviving individuals were euthanized and underwent a sectional and microbiological examination. The presence and number of *E. coli* in the ileum, cecum, and colon was evaluated after streaking plates of G2SN with intestinal contents and incubating aerobically at 37 °C for 18 h, G2SN medium. Five colonies from each section were characterized in terms of belonging to serogroup O103 using standard methods. 

### 2.9. Statistical Analysis

Results obtained were subjected to statistical analysis using the STATISTICA 10.0 software (Statistica, Tulsa, OK, USA). The data are presented as average values, accompanied by standard error of the means and standard deviation. Data were analyzed using a general linear model for repeated measures two-way ANOVA with dietary treatments (*D*) and sampling time (*T*) as fixed effects and their interactions (*D* × *T*). Differences between treatment group means were analyzed for significance (*p* < 0.05) using the Tukey post hoc test. 

## 3. Results and Discussion 

Amounts of chemical fractions analyzed in herbs as well as their water macerates are presented in Table 1. Spectrophotometric determination of fractions applied in experimental plants gave the expected results. Highest content of tannins was assessed in *P. anserine* 78.1 and 54.2 in *P. aviculare*, whereas in inflorescence of *R. crispus* we found only 16 mg/g. Similar values (from 40 to 101 mg/g) were observed by Lincheva et al. [22] in Bulgarian samples of *Potentilla*. In polish cultivars of *P. anserine*, spectrophotometric determination allowed the finding of 95.2 mg/g d.m. of tannins in herbs [35]. Grujić-Vasić et al. [8] found 2.7% of tannins in roots of the Balkanian variety of *Potentilla*, and moderate antimicrobial activity against *Staphylococcus aureus* strain ATCC6538 T was proven. Interestingly, water extracts of *Potentilla* sp. cultivated in China and rich in tannins have been shown to strongly inhibit the growth of *Pseudomonas aeruginosa* and *Candida albicans,* whereas *E. coli* strains were resistant [36]. Amount of tannins in *P. aviculare*, 54.2 mg/g d.m., were similar to the values described (45 mg/g) in EMA monographies [5]. There is no available information about the chemical composition of *R. crispus* inflorescence, although in dry fruits Wagiera et al. [37] found much higher percentage of tannins at 4.8% compared to our results using the spectrocolorimetric method. Those authors assessed only 0.021% of anthracene derivatives and 0.02% of polyphenols. Wagiera et al. [36] also determined the antimicrobial activity of etanolic extracts with a minimal inhibitory concentration on range 125–>500 μg/mL for *Escherichia coli ATCC 3521, Proteus mirabilis, and Pseudomonas aeruginosa*. According to the above facts, it is reasonable to conclude that those three herb extracts prevent bacterial infection.

The mixture of herbs which included Common silverweed (*Pontentilla anserina*), Common knotgrass (*Polygonum aviculare*) and Rumex nervosus was used in the study. The recipe resulted from the previously proven effect of these herbs consisting in the reduction of the frequency and severity of diarrhea symptoms [20] as well as reports of natural medicine and the above-described antibacterial properties. Common silverweed (*Pontentilla anserina*) was used in traditional medicine to treat inflammation, infections caused by bacteria, fungi, and viruses and to treat diarrhea [33]. Common knotgrass (*Polygonum aviculare*) shows, inter alia, positive effects on wound healing, intestinal epithelial reconstruction and antibacterial properties [37]. The approximately 200 species of the genus *Rumex* are distributed worldwide in European, Asian, African, and American countries [38]. Some species have been used traditionally as vegetables and for their medicinal properties. In vitro studies demonstrated that Rumex nervosus has a marked bactericidal and bacteriostatic activity [2]. The anti-inflammatory activity test results verified that *R. abyssinicus* inhibited the synthesis of PGE2 [3]. This and the antibacterial activity of the plant may justify its traditional use for the treatment of several skin diseases. 

The growth performance of rabbits from the start of supplementation to 80 days old is shown in Table 2. No significant differences were found between the groups. The data showed that daily feed intake and daily body weight gain were higher than those of the control group after including the herb in the feed. In the ExpW group, daily body weight gain was the lowest. Regardless of the method of herb administration, the experimental group rabbits consumed less water than the control group.

Table 3 presents the mean values of hematological indices of rabbit blood. There were no statistical differences in WBC between the groups, while differences were related to the time of herbal supplementation (*p* < 0.01). A significant (*p* < 0.01) effect of supplementation and time on the mean RBC was found. RBC was the highest in the ExpF group. There were no significant differences in the mean values of HGB and HCT. The platelet count was higher in the experimental groups (*p* < 0.03) than in the control group. At the end of the study, PLT was the highest in the ExpW group, taking into account the date of blood sampling. A significant effect of the diet on MCH and MCHC was observed (Table 3). The values noted in this study were similar or slightly higher than those reported in other studies [20,39]. Normalization of hematological parameters in rabbits with diarrhea was found in a previous study after an application of ethanol extract of propolis and a mixture of herbs [20]. The current experiment additionally indicates that the inclusion of *Rumex cissus*, *Pontentilla anserine,* and *Polygonum aviculare* in the water leads to a linear increase in WBC. This effect was less visible when these herbs were added to the feed.

No acid-base balance disturbances were found in all groups during the experimental period (Table 4). The interpretations were based on reference values given by Ardiaca et al. [40]. Blood pH was lower in the ExpW and ExpF groups compared to the control one, and these changes were significantly related to time (*p* < 0.04). Concurrently, the mean values of pCO_2,_ HCO_3_^-^ and BE were lower in the ExpW group compared to the ExpF and control group. In the case of pCO_2_ and HCO_3_^-^, the differences between the groups were statistically significant considering the term of analyses (*p* < 0.01). Depending on the time of supplementation, the growth of the metabolic component (HCO_3_^−^ BE) occurred in the ExpF group. The obtained results indicate that the applied herbs affect acid-base balance, and this effect depends on the method of their administration. There was a significant interaction of time and diet in the case of Na and K cations concentration (*p* < 0.03). The mean values were the highest at the end of supplementation in both experimental groups. The concentration of these cations was the lowest in the ExpW group, and the highest in ExpF. The applied diet had a significant (*p* < 0.03) effect on an occurrence of differences in Cl anions concentration. 

The administration of herbs caused an increase in the mean concentration of TP and albumin in the blood serum (*p* < 0.01); also, the date of sampling had a significant effect on the differences between the groups (*p* < 0.01) (Table 5). The concentration of TP and serum albumin was the highest in the ExpW group. Significantly higher AST and GGT activity was found in the ExpW and ExpF groups compared to the control group. AST activity was also affected by the time of supplementation since the activity of this enzyme was found to increase in the last blood sampling. The highest concentration of bilirubin and GGT activity were recorded in the ExpF group. Despite an increase in liver enzyme activity in all studied groups, they remained within the normal range [40], which indicates that the extract of herbs in the tested doses did not adversely affect the secretory functions of the liver. 

The herbs used in this study did not affect blood cholesterol level. A significant (*p* < 0.05) reduction in blood cholesterol level was noted in another study in rabbits with prolonged pituitrin hypertension that orally received a mixture made from herbal preparation [40]. The two different methods of herbs supplementation used in this study, despite some differences in the concentration of biochemical parameters in the blood, did not cause an excessive proteolytic catabolism or liver dysfunction. Unfavorable intensification of these changes is observed in the course of diarrhea [20,41]. In addition, no increased lipolysis was found, which confirms the lack of additives’ effect on feed consumption and lipid metabolism. The applied supplementation and time had a significant effect on glucose concentration (*p* < 0.01); the lowest concentration was recorded in the ExpW group. There was a significant effect of the date of blood sampling on LA and total cholesterol concentration (*p* < 0.01), but no effect of treatment was noted. 

The highest total antioxidant assay (TAS) value was observed in the ExpF group, which was significantly associated with the supplementation used (*p* < 0.01). TAS enables us to evaluate the entire antioxidant system, which includes all active biological components showing activity in the prevention of the negative effects of free radicals [42]. Therefore, its higher values in the groups receiving herbs indicate the effect of active substances contained in herbs with antioxidant effects [42]. A number of plants exhibit protective activity against reactive oxygen species. Supplementation with tannin and gallic acids from *Rhus coriaria* in rabbits causes TAS increase [43]. Importantly, this study showed a significant dose-dependent effect. The fact of an effect of the herbs used on the antioxidant status is confirmed by GPx activity. The highest GPx activity was recorded in the control group and the lowest in the ExpF group (Table 5). Differences in GPx activity were related to the diet (*p* < 0.01) and blood sampling time (*p* < 0.03). GPx is one of the most important enzymes involved in the elimination of intracellular free radical. In the case of GPx, the activity is significantly dependent on the nutritional factor. It was observed in the study by Larocca et al. [44] that LPS injection in rabbits causes a decrease in the activity of a number of antioxidant enzymes (e.g., GSH, SOD, and CAT), while prophylactic supplementation of *Brassica* phytochemicals increases the activity of these antioxidant enzymes. In addition, albumins represent a very abundant and important circulating antioxidant. In our study, the highest concentration was found in ExpW and then in ExpF. 

In rational rabbit breeding units of Western Europe, colibacillosis has become one of the most economically and pathologically important diseases. Since the beginning of the 1980s [45], pathogenicity mechanisms of EPEC are now well understood, while the intrinsic and environmental factors that control the expression of EPEC virulence remain largely unknown [46]. The O103 eae + strain was used in the study due to strong pathogenicity for rabbits. Serogroup O 103 is one of the most frequently isolated from rabbit colibacillosis cases; this strain is considered highly pathogenic for post-wean rabbits [47,48]. In an experimental infection of the control group (n = 6), 2 rabbits died on days four and six (Table 6). All rabbits from this group (both dead and euthanized) showed typical sectional changes for colibacteriosis: Fluid contents in the small and large intestines and petechiae in the caecum in dead individuals. In the case of experimental groups treated with herbs and infected (n = 6), the number of falls was one in each group. Sectional changes were typical for colibacillosis (fluid content in the small and large intestines). 

Microbiological examination of experimental animals is presented in Table 7. In the case of rabbits from uninfected groups, the number of *E. coli* in individual sections varied from 6.1 x 10^3^ to 7.2 × 10^3^, with the lowest number found in small intestines and the largest in the colon in all groups. These results coincide with the results obtained by other authors [49,50]. 

In the case of infected rabbits in the ExpP group, the highest number of *E. coli* was found in cecum. Comparing the number of *E. coli* in individual sections of the intestine, between infected and uninfected groups, an effect of herbs on its reduction was found. Administration of a mixture of herbs in water similarly reduced the number of *E. coli* in ileum. Experimentally infected rabbits from the ExpW group had as much as 8.7 × 10^7^ *E. coli* in ileum. A lower number of *E. coli* in particular intestinal segments was found in the ExpF group. Summing up the results of the study, it should be concluded that irrespective of the method of herbal extract application, a lower number of rabbits fell into the treated groups. However, regarding intestine colonization by *E. coli*, a significant decrease in the number of bacteria was found in ileum, cecum, and colon after the use of the herbal extract in feed or water. Limited growth of enteropathogenic bacteria (*E. coli* O103 strain) and a lower number of falls was found in the case of pure caprylic acid and triacylglycerols of both caprylic and capric acid in rabbits with diarrhea [50]. In another study [51], in gnotobiotic mice with EHEC O157 strain-induced infection, green tea extract acted synergistically to antibiotic treatment. The plant extracts may be a good alternative to antibiotic treatment or may be complementary to them especially due to the risk of more severe clinical conditions resulting from the use of antibiotics [52]. Experimental supplementation with medium-chain fatty acids (MCFA) did not reduce the sensitivity of rabbits challenged with EPEC strain [46]. Anti-inflammatory activity was found applying powder from cauliflower leaves (*Brassica oleracea* var. *botrytis*) before injection of LPS [44]. This study points out that the form of supplementation may be a significant issue in case of herbal extracts.

## 4. Conclusions

The herbs applied in rabbits did not have a negative impact on the secretory functions of the liver, electrolyte, and acid-base balance of the blood. An effect on hematological and biochemical parameters was differentiated. Undoubtedly, the applied supplementation affected the oxidative status; TAS was the highest in the ExpF group. The rabbit model used during the experimental infection with *E. coli* indicates a clear effect of *Rumex crispus* L., *Pontentilla anserina*, and *Polygonum aviculare*, and the activity involves the reduction of colonization of basically all sections of the intestines. The use of herbs in rabbits can control the activity of the intestinal microbial community. This suggested that the herbal combination could serve as a safe component in the treatment of EPEC infection. Further studies should clarify the use of the other herbal combination as an associated treatment for EPEC infections in animal model.

## Figures and Tables

**Table 1:** Analyzed fractions in herbs and macerates.

	TPC ^a^		Tannins ^b^		Protoanthocyanidines ^c^		Saponins		Antraquinones	
	mg/g ^d^	mg/mL ^e^	mg/g ^d^	mg/mL ^e^	mg/g ^d^	mg/mL ^e^	mg/g ^d^	mg/mL ^e^	mg/g ^d^	mg/mL ^e^
*P. anserine*	125.1 ± 23	2.74 ± 0.23	78.1 ± 13.40	1.68 ± 0.15	95.12 ± 7.30	2.21 ± 0.12	18.6 ± 4.10	0.19 ± 0.05	n.d.	n.d.
*P. aviculare*	106.8 ± 12	2.57 ± 0.15	54.2 ± 7.31	1.02 ± 0.12	69.1 ± 11.10	1.19 ± 0.09	39.4 ± 6.20	0.42 ± 0.02	n.d.	n.d.
*R. crispus*	39.6 ± 8.1	0.96 ± 0.18	16.3 ± 2.50	0.51 ± 0.10	47.8 ± 9.30	1.09 ± 0.08	n.d.	n.d.	2.1 ± 0.47	0.05 + 0.02

^a^ Total phenolic content expressed as mg of GA; ^b^ expressed as TAE; ^c^ expressed as TAE; ^d^ measured and expressed as mg of compounds in 1 g of dried plant material; ^e^ measured and expressed in 1 mL of used macerate; n.d.—not detected, SD on the basis of three replicates.

**Table 2:** Growth performance and water consumption of rabbits.

Item *	Group			SEM	*p*-Value
	Control	ExpW	ExpF		
Daily body weight gain (g)	35.1	33.8	36.3	1.76	0.08
Daily feed intake (g)	160	160.3	164.3	12.56	0.23
Feed conversion rate (g/g)	4.6	4.7	4.5	0.09	0.06
Water consumption (mL/d)	305.5	292.6	296.4	12.25	0.09

* During period of treatment with herbs (50–80 d of live).

**Table 3:** Mean values of hematological parameters in rabbit blood.

Item	Group			SEM	*p*-Value		
	Control	ExpW	ExpF		D	T	Interaction
							T × D
WBC, G/L	6.80 ^a^	7.13 ^b^	6.67	0.22	0.15	<0.01	0.27
RBC, T/L	5.58 ^A^	5.64	5.80 ^B^	0.03	0.01	0.01	0.11
HGB, mmol/L	7.27	7.38	7.31	0.04	0.64	0.57	0.29
HCT, l/l	0.38	0.38	0.39	0.01	0.64	0.56	0.38
PLT, G/L	357.54	405.2	366.89	17.03	0.48	0.03	0.44
MCV, fL	68.37 ^A^	68.03 ^A^	66.83 ^B^	0.3	0.01	<0.01	0.14
MCH, fmoL	1.30 ^A^	1.31 ^A^	1.26 ^B^	0.01	<0.01	<0.01	0.1
MCHC, mmol/L	19.03	19.19	18.89	0.04	0.09	0.21	0.12

*p*-value—significant effect of experimental diet (D), time on diet (T), and their interaction (D × T); SEM—standard error of the means. ^A, B^—Means in rows marked with different lowercase superscripts differ significantly at *p* < 0.01; ^a, b^—Means in rows marked with different lowercase superscripts differ significantly at *p* < 0.05.

**Table 4:** Mean values of acid-base balance and electrolyte parameters in rabbit blood.

Item	Group			SEM	*p*-Value		
	Control	ExpW	ExpF		D	T	Interaction
							T × D
pH	7.48	7.46	7.46	0.01	0.06	0.04	0.94
pCO_2_, kPa	4.72 ^A^	4.61 ^B^	4.96 ^A^	0.01	0.05	0.01	0.05
HCO_3_^-^, mmol/L	24.57	22.67	24.26	0.41	0.14	0.01	0.19
BE, mmol/L	2.34 ^A^	0.48 ^B^	0.67 ^B^	0.36	0.06	0.08	0.1
Na^+^, mmol/L	138.31	136.76	139.5	0.01	0.01	0.02	0.03
K^+^, mmol/L	4.92	4.79	5.01	0.06	0.35	<0.01	0.03
Cl^-^, mmol/L	103.07	101.98	112.56	0.83	0.03	0.08	0.24

*p*-value—significant effect of experimental diet (D), time on diet (T), and their interaction (D × T); SEM—standard error of the means. ^A, B^—Means in rows marked with different lowercase superscripts differ significantly at *p* < 0.01.

**Table 5:** Mean values of biochemical blood parameters in rabbit blood, TAS, and GPx activity.

Item	Group			SEM	*p*-Value		
	Control	ExpW	ExpF		D	T	Interaction
							T × D
pH	7.48	7.46	7.46	0.01	0.06	0.04	0.94
pCO_2_, kPa	4.72 ^A^	4.61 ^B^	4.96 ^A^	0.01	0.05	0.01	0.05
HCO_3_^−^, mmol/L	24.57	22.67	24.26	0.41	0.14	0.01	0.19
BE, mmol/L	2.34 ^A^	0.48 ^B^	0.67 ^B^	0.36	0.06	0.08	0.1
Na^+^, mmol/L	138.31	136.76	139.5	0.01	0.01	0.02	0.03
K^+^, mmol/L	4.92	4.79	5.01	0.06	0.35	<0.01	0.03
Cl^−^, mmol/L	103.07	101.98	112.56	0.83	0.03	0.08	0.24

*p*-value—significant effect of experimental diet (D), time on diet (T), and their interaction (D × T); SEM—standard error of the means. ^A, B, C^—Means in rows marked with different lowercase superscripts differ significantly at *p* < 0.01.

**Table 6:** Mortality of rabbits after experimental *E. coli* infection (strain O103 *eae*^+^).

Group	Number of Animals Fallen after Infection	Number of Animals Subjected to Euthanasia	Section Changes
Control (n = 6) *uninfected*	0	6	No changes
Control (n = 6) *infected*	2 (4 and 6 day)	4	Typical for E. coli *
ExpW (n = 6) *uninfected*	0	6	No changes
ExpW (n = 6) *infected*	1 (5day)	5	Typical for E. coli *
ExpF (n = 6) *uninfected*	0	6	No changes
ExpF (n = 6) *infected*	1 (5 day)	5	Typical for E. coli *

* Typical for *E. coli* symptoms: bloating of the gastrointestinal tract, fluid content in the gastrointestinal tract, bloody ecchymosis in the serosa of the large intestine.

**Table 7:** Effect of herbs supplementations on number of *E. coli* in different part of gastrointestinal (cfu/g).

Group	Number of *E. coli*		
	Ileum	Cecum	Colon
Control (n = 6) *uninfected*	6.1 × 10^3 B^	8.1 × 10^3 A^	4.9 × 10^4 A^
Control (n = 6) *infected*	1.1 × 10^8 A^	6.2 × 10^8 B^	4.7 × 10^9 B^
ExpW (n = 6) *uninfected*	4.8 × 10^3 B^	6.7 × 10^3 AC^	5.4 × 10^4 AB^
ExpW (n = 6) *infected*	8.7 × 10^7 Aa^	3.1 × 10^8 D^	2.6 × 10^9 C^
ExpF (n = 6) *uninfected*	4.7 × 10^3 bB^	7.2 × 10^3 AE^	4.9 × 10^4 AD^
ExpF (n = 6) *infected*	6.4 × 10^4 B^	2.7 × 10^8 BF^	1.3 × 10^9 BD^

^A, B, C, B, E, F^—Means sharing the same letter in each column are not significantly different at *p* < 0.01. ^a, b^—Means sharing the same letter in each column are not significantly different at *p* < 0.05.

## Supplementary Materials

The following are available online at https://www.mdpi.com/2076-2615/9/10/774/s1, Table S1: Profile and content of Rumex inflorescence fatty acids, Table S2: GC-MS profile and content of Rumex polar fraction.
