# Supplementary material for: Application of Pontentilla Anserine, Polygonum aviculare and Rumex Crispus Mixture Extracts in A Rabbit Model with Experimentally Induced E. coli Infection
Source: Animals (Basel). 2019 Oct 9;9(10):774. doi: 10.3390/ani9100774 (PMC6826411; doi:10.3390/ani9100774)
Supplement: Supplementary file 1 [file animals-09-00774-s001.pdf]

Table S1. Profile and content of *Rumex* inflorescence fatty acids

nr	tR (min)	Peak Name	mg/g
1	24.435	Methyl tetradecanoate	0.25
2	27.065	Pentadecanoic acid, methyl ester	0.13
3	27.565	5-Octadecenoic acid, methyl ester	0.17
4	29.620	Hexadecanoic acid, methyl ester	13.16
5	30.600	Methyl hexadec-9-enoate	0.28
6	32.035	Heptadecanoic acid, methyl ester	0.18
7	32.880	cis-10-Heptadecenoic acid, methyl ester	0.13
8	34.425	Methyl stearate	2.20
9	35.175	Oleic acid, methyl ester	42.07
10	35.310	Elaidic acid, methyl ester	2.56
11	36.505	Linoleic acid, methyl ester	35.29
12	37.870	Linolenic acid, methyl ester	4.53
13	38.230	Eicosanoic acid, methyl ester	1.00
14	38.655	cis-Methyl 11-eicosenoate	1.39
15	40.795	Docosanoic acid, methyl ester	0.77
16	41.100	13-Docosenoic acid, methyl ester, (Z)-	2.76
17	42.760	Tetracosanoic acid, methyl ester	1.11
18	43.005	5-Tetracosenoic acid, methyl ester, (Z)-	1.19
19	44.585	Pentacosanoic acid, methyl ester	0.83
Total			109.5

Table S1. GC-MS profile and content of Rumex polar fraction

Nr	tR (min)	Peak Name	Area (%)
1	6.053	Malic acid, 3TMS derivative	1.975
2	6.847	5-Oxoproline, , 2TMS derivative	0.827
3	8.053	Erythronic acid TMS derivative	0.508
4	11.622	Xylose TMS derivative	0.320
5	15.100	Ribose, 4TMS derivative	3.108
6	18.850	Shikimic acid, 4TMS derivative	2.451
7	19.373	Citric acid, 4TMS derivative	0.294
8	20.252	Ribofuranose	0.408
9	22.272	Quininic acid, TMS derivative	3.505

10	23.342	Fructose Isomer 1	13.877
11	24.050	Fructose Isomer 2	12.202
12	24.920	Galactose Isomer 1	10.484
13	25.950	Galactose, Isomer 2	2.339
14	26.542	Gallic acid, 4TMS derivative	0.240
15	26.967	unknown	0.349
16	35.743	Myo-Inositol, 6TMS derivative	9.377
17	36.935	Inositol, epi-, 6TMS derivative	0.475
18	37.692	Allopyranose	0.312
19	37.820	unknown	0.430
20	48.608	Sucrose, 8TMS derivative	20.031
21	50.150	Trehalose, TMS derivative	1.019
22	51.407	Catechine, TMS derivative	1.376
23	51.783	Catechine isomer, TMS derivative	11.869
24	53.673	Galactinol, TMS derivative	0.214
25	55.577	CAS nr 6651 - 66 - 7	1.041
26	55.772	Quercetin, TMS derivative	0.969

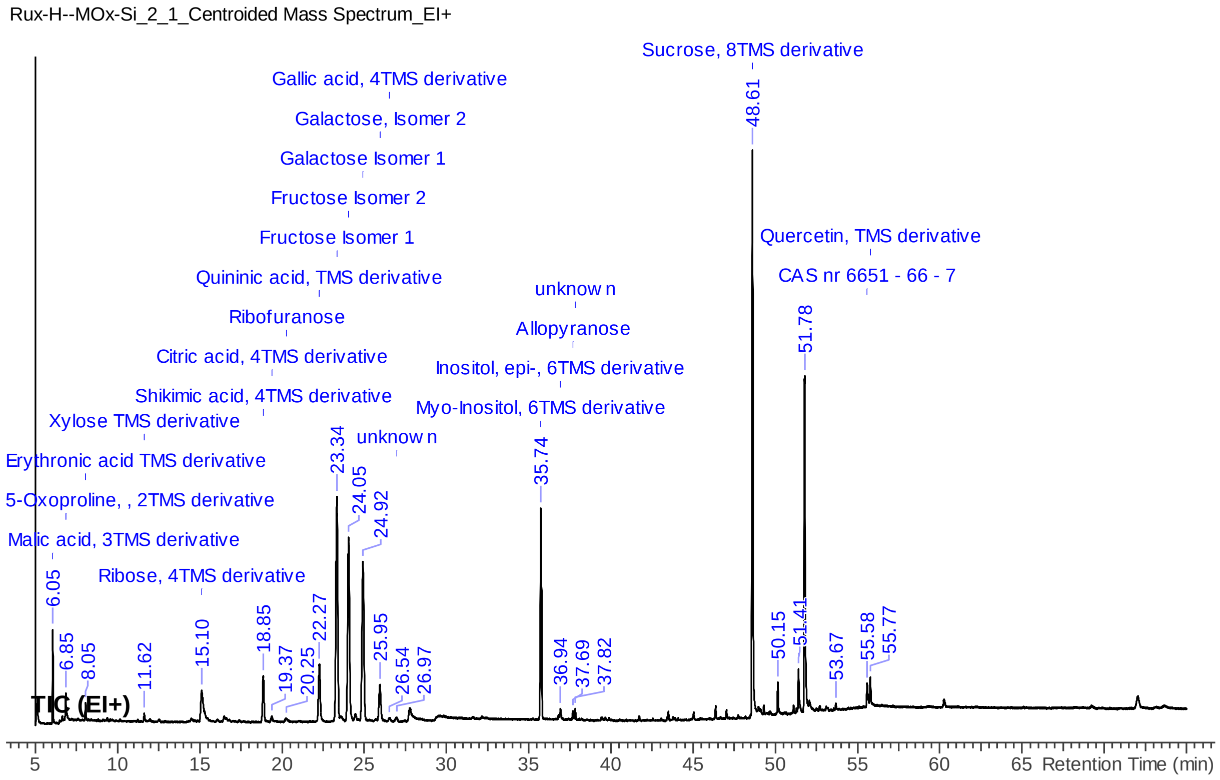

Figure 1. GC-MS profile of Rumex polar ftaction

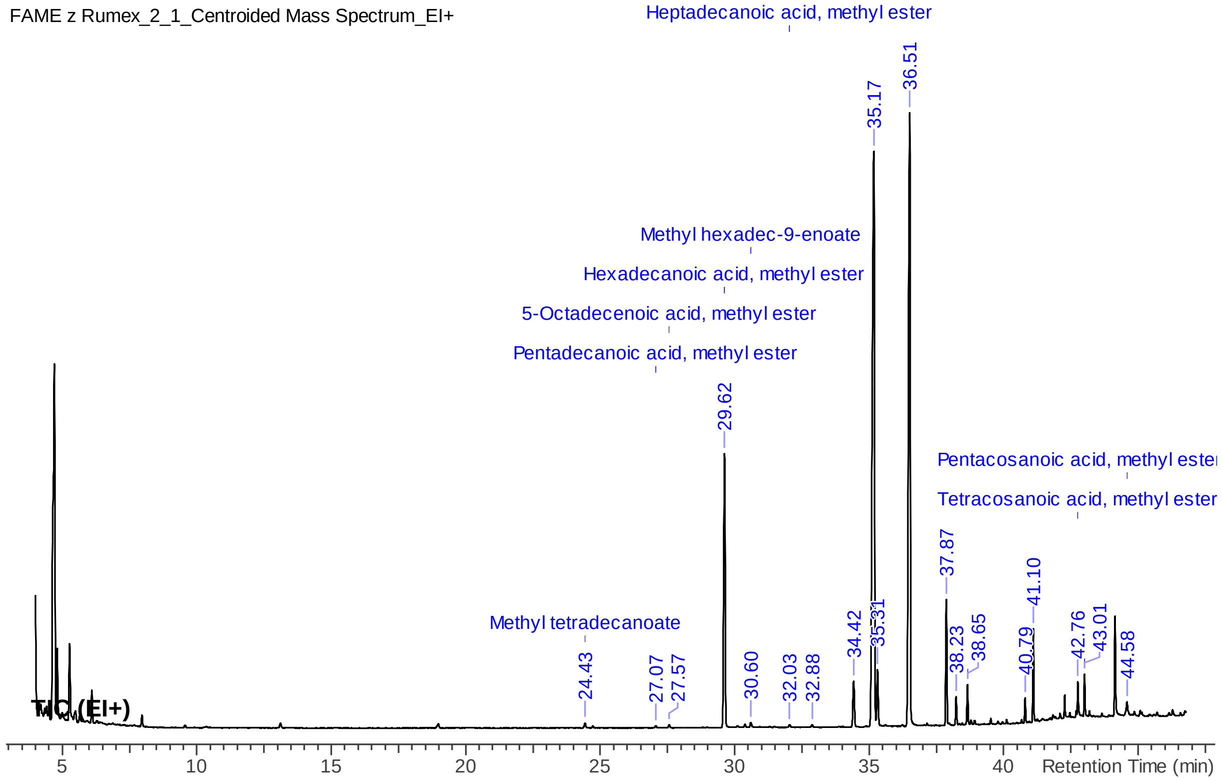

Figure 2. GC-MS profile of Rumex lipid ftaction

Table 3. Volatile composition of *R. crispus* inflorescence

No	tR (min)	Peak Name	KI exp.	KI lit.	Percentage
1	3.76	Hexanal	801	802	2.33
2	4.752	2-Hexenal, (E)-	857	854	1.65
3	4.823	3-Hexen-1-ol, (Z)-	861	857	2.76
4	5.076	1-Hexanol	869	868	2.43
5	5.849	Heptanal	900	901	1.55
6	7.34	2-Heptenal, (Z)-	960	958	4.12
7	7.509	Benzaldehyde	970	962	2.29
8	7.733	1-Heptanol	972	970	1.00
9	7.861	3-octen-2-ol, (3Z)-	976	978	1.16
10	8.017	1-Octen-3-one	981	979	5.52
11	8.243	5-Hepten-2-one, 6-methyl-	987	986	3.63
12	8.411	Furan, 2-pentyl-	992	993	1.96
13	8.565	2-octanol	997	995	2.46
14	8.735	Octanal	1001	1003	3.79
15	9.002	2-hexen-1-ol, acetate, (2E)-	1011	1012	3.03
16	9.694	3-octen-2-one (3E)-	1035	1034	0.44
17	10.074	Benzeneacetaldehyde	1045	1045	1.52
18	10.384	unknown	1052		1.75
19	10.523	2-Octenal, (E)-	1053	1060	2.34
20	10.636	unknown	1062		3.22
21	10.861	5-Octen-1-ol, (E)-	1070	1171	1.61
22	10.959	5-Octen-1-ol, (Z)-	1074	1172	4.17
23	11.241	unknown	1083		1.67
24	12.079	Nonanal	1099	1104	14.05
25	12.416	Phenylethyl Alcohol	1114	1116	4.40
26	13.804	2,6-Nonadienal, (E,Z)-	1150	1154	0.96
27	13.973	2-Nonenal, (E)-	1158	1160	1.40
28	14.393	1-Nonanol	1174	1173	1.39
29	15.55	Decanal	1206	1206	3.19
30	16.092	β-Cyclocitral	1219	1220	2.54
31	17.449	2-Decenal, (E)-	1263	1263	4.19
32	18.52	2-Undecanone (IS)	1294	1296	-
33	18.959	Undecanal	1306	1307	1.18
34	21.86	acetic acid, nonyl ester	1312	1314	tr.
35	20.745	2-Undecenal	1369	1369	1.50
36	22.808	trans-Geranylacetone	1458	1453	2.44
37	23.488	β-Ionone	1491	1491	1.07
38	24.18	Actinidiolide, dihydro-	1533	1532	1.87

39	25.266	Tetradecanal	1618	1620	0.71
40	26.138	(E)-Tetradec-2-enal	1670	1673	2.70

KI exp. – Experimental Kovats retention index calculated on the basis of series n-alkanes C-8 to C-20;

KI lit. – literature Kovats retention index (NIST 14 database); tr: less than 0.05%

Table 4. Sterol profile of inflorescence of *Rumex crispus*

No.	Peak Name	tR (min)	mg/g
1	Cholesterol (IS)	26.085	-
2	Unknown	26.365	1,923594
3	Desmosterol	26.595	2,417463
4	Lanosterol	26.850	2,703155
5	Campesterol	27.535	4,635301
6	Stigmasterol	27.985	3,177721
7	beta-Sitosterol	28.905	5,369667
Total			20,2269
